# Partial Block by Riluzole of Muscle Sodium Channels in Myotubes from Amyotrophic Lateral Sclerosis Patients

**DOI:** 10.1155/2014/946073

**Published:** 2014-12-08

**Authors:** Cristina Deflorio, Emanuela Onesti, Clotilde Lauro, Giorgio Tartaglia, Aldo Giovannelli, Cristina Limatola, Maurizio Inghilleri, Francesca Grassi

**Affiliations:** ^1^Department of Physiology and Pharmacology, Pasteur Institute-Cenci Bolognetti Foundation, Sapienza University, Piazzale Aldo Moro 5, 00185 Rome, Italy; ^2^Department of Neurology and Psychiatry, Sapienza University, Viale dell'Università 30, 00185 Rome, Italy; ^3^Department of Applied Clinical and Biotechnological Sciences, University of L'Aquila, Via Vetoio, Coppito 2, 67100 L'Aquila, Italy; ^4^IIT@Sapienza, Center for Life Nano Science, Viale Regina Elena 291, 00161 Roma, Italy; ^5^IRCCS Neuromed, 86077 Pozzilli, Italy

## Abstract

Denervated muscles undergo fibrillations due to spontaneous activation of voltage-gated sodium (Na^+^) channels generating action potentials. Fibrillations also occur in patients with amyotrophic lateral sclerosis (ALS). Riluzole, the only approved drug for ALS treatment, blocks voltage-gated Na^+^ channels, but its effects on muscle Na^+^ channels and fibrillations are yet poorly characterized. Using patch-clamp technique, we studied riluzole effect on Na^+^ channels in cultured myotubes from ALS patients. Needle electromyography was used to study fibrillation potentials (Fibs) in ALS patients during riluzole treatment and after one week of suspension. Patients were clinically characterized in all recording sessions. In myotubes, riluzole (1 *μ*M, a therapeutic concentration) reduced Na^+^ current by 20%. The rate of rise and amplitude of spikes evoked by depolarizing stimuli were also reduced. Fibs were detected in all patients tested during riluzole treatment and riluzole washout had no univocal effect. Our study indicates that, in human myotubes, riluzole partially blocks Na^+^ currents and affects action potentials but does not prevent firing. In line with this *in vitro* finding, muscle Fibs in ALS patients appear to be largely unaffected by riluzole.

## 1. Introduction

The current criteria for diagnosis of amyotrophic lateral sclerosis (ALS) require neurophysiological evidences of ongoing denervation, defined by fibrillation potentials (Fibs) or positive sharp waves, and of chronic partial reinnervation, involving enlarged, unstable motor units with a reduced interference pattern [1]. Fibs are detected as long as denervation-reinnervation processes take place [2], and their assessment is considered a sensitive method to evaluate disease progression and a useful research tool (see, for instance, [3]). At later stages of disease they fade owing to replacement of muscle fibres with fibrous tissue.

In mammalian denervated muscle fibres, fibrillations arise because spontaneous activation of voltage-gated Na^+^ (Na_V_) channels triggers action potentials and contractions. Accordingly, only Na_V_ channel blockers stop fibrillations* in vitro* [4].* In vivo*, clinically used doses of lidocaine blocked Fibs in denervated rats [5]. Disappearance of Fibs has been reported in one ALS patient receiving fentanyl and propofol during surgery [6], two anaesthetics that block Na_V_ channels [7, 8].

Riluzole is a well-established blocker of neuronal Na_V_ channels, but it also affects other channels in nonneuronal tissues [9], for instance, muscle acetylcholine receptor channels [9–12]. Riluzole also causes a mild block of recombinant and native muscular voltage-gated Na^+^ current (*I*_Na_) [10, 13], although at concentrations higher than those attained in patients' plasma [9]. In myotubes, riluzole reduces the frequency of (rarely occurring) spontaneous contractions [13]. Given these premises, riluzole might affect Fibs in ALS patients, but this point has received little attention to date, even if riluzole is the only approved drug for ALS treatment [14]. Therefore, we undertook a study of riluzole action on Fibs, aimed at filling this gap.

Detection of Fibs in patients requires needle electromyography with uncomfortable multiple insertions. Thus the study was started* in vitro.* Human muscle satellite cells can be cultured* in vitro* in the absence of nerve, and myotubes adequately represent denervated muscle fibres [15]. Moreover, satellite cells* in vitro* preserve traces of their* in vivo* environment [16] including denervation-induced alterations [17]. Satellite cells derived from ALS patients display a reduced myogenic potential [18] but form multinucleated myotubes (ALS myotubes; [11]). As commonly observed for primary human myotubes [19–21], ALS myotubes do not contract spontaneously. In the context of Na_V_ channels, both denervated muscle fibres and cultured myotubes express Na_V_1.5 channels in addition to Na_V_1.4, the only form present in innervated muscle fibres [22, 23]. Thus, ALS myotubes provide a valuable experimental model with functional properties conditioned by donor health status. We therefore first evaluated the effect of riluzole on muscle *I*_Na_ and action potentials* in vitro* and then verified our conclusions* in vivo*, in a group of patients.

In ALS myotubes, riluzole affected the amplitude and voltage dependence of *I*_Na_. As a consequence, spike amplitude and rate of rise were reduced, but action potential firing was not suppressed, suggesting that riluzole would not prevent muscle fibrillations. Indeed, Fibs were routinely detected during riluzole administration in ALS patients and brief suspension of the treatment had no univocal effects on Fibs frequency. All our data suggest that riluzole, in spite of partially blocking muscle Na_V_ channels, does not interfere with muscle Fibs.

## 2. Materials and Methods

### 2.1. Cell Culture

Human myotubes were grown from satellite cells derived from muscle biopsies performed at the ALS Centre of Policlinico Umberto I, Sapienza University. All patients gave written informed consent to use part of the biopsy material for the research project entitled “Study of Nicotinic Acetylcholine Receptor in Muscle Tissue of ALS Patients,” approved by the Human Subjects Ethical Committee of Policlinico Umberto I. Satellite cells were obtained immediately after biopsy as described [11, 12]. Frozen aliquots were thawed for the present study and propagated as previously reported [11, 12]. Differentiation was induced at 50% confluence by switching to low-serum differentiating medium (Dulbecco's minimum essential medium plus 2% horse serum and penicillin/streptomycin). Experiments were performed 3 to 8 days after medium switch.

### 2.2. Patch-Clamp Recordings and Data Analysis

Whole-cell patch-clamp recordings were made at room temperature (23–27°C) using an Axopatch 200B amplifier (Molecular Devices, Union City, CA, USA), driven by pClamp 9 (Molecular Devices). Cells were continuously superfused using a gravity-driven fast exchanger perfusion system (RSC-200, Bio-Logic, France). External solution contained (mM) 140 NaCl, 2.8 KCl, 2 CaCl_2_, 2 MgCl_2_, 10 HEPES-NaOH, and 10 glucose, pH 7.3. Tetrodotoxin (TTX) and riluzole were added as indicated. In some experiments, addition of 4-aminopyridine (2 mM) to block voltage-gated K^+^ channels did not affect *I*_Na_. Patch pipettes (2–5 MΩ) contained (mM) 130 CsCl, 10 NaCl, 5 BAPTA, 10 HEPES-KOH, 2 Mg-ATP, and 2 MgCl_2_, pH 7.3. To record action potentials, KCl was used instead of CsCl. All salts were from Sigma Italia (Milano, Italy). Recordings were considered only if patch series resistance was less than 8 MΩ and compensated by 95–99%. Riluzole-induced shifts of activation and inactivation curves were unaltered if voltage drop over uncompensated access resistance (*R*_*U*_) was kept into account correcting the test potential (*V*_*m*_) as *V*_Corr_ = *V*_*m*_ − *R*_*U*_∗*I*_Na_ and using *V*_Corr_ in data analysis.

Current-voltage curves were obtained applying membrane potential steps (5 ms at 1 s interval) in 5 mV increments from −65 mV to +60 mV, from a steady holding potential of −80 mV. Fast inactivation curve was obtained using a two-pulse protocol: prepulse potential (20 ms) was changed from −140 mV to −15 mV in 5 mV increments, followed by a test pulse to −10 mV (5 ms at 1 s interval), as previously done in human muscle fibres [24]. Data were filtered at 5 kHz and digitized at 50 kHz.

Current reversal potential (*E*_Na_), estimated from plot of peak *I*_Na_ *versus V*_*m*_, was 64 ± 1 mV (*n* = 30), as predicted by Nernst equation (66 mV). Conductance was calculated as *G* = *I*_Na_/(*V*_*m*_ − *E*_Na_) and normalized to the maximal value *G*_max⁡_. Values of *G*/*G*_max⁡_ were then fitted with Boltzmann equation:

(1) $$\begin{array}{c} \frac{G}{G_{\max}}=\frac{1}{\left[1+e^{\left(V-V_{A}\right)/k_{A}}\right]}, \end{array}$$

where *V*_*A*_ is the potential at which conductance is half of maximal value and *k*_*A*_ is the slope factor. Fast inactivation relationships were constructed plotting current amplitude *I*, normalized to maximal value *I*_max⁡_,* versus* prepulse potentials and fitting a Boltzmann equation to data points:

(2) $$\begin{array}{c} \frac{I}{I_{\max}}=\frac{1}{\left[1+e^{\left(V-V_{I}\right)/k_{I}}\right]}, \end{array}$$

where *V*_*I*_ is the potential at which current is half of maximal value and *k*_*I*_ is the slope factor.

Action potentials were recorded under current clamp conditions using stimuli of increasing amplitude (from 0.2 to 1 nA) at 1 s intervals. Phase plots, representing changes in membrane potential with time (i.e., the first derivative of membrane potential)* versus* the value of membrane potential at the corresponding time, were built for each action potential [25].

Results are given as mean ± SEM. Two data sets were considered statistically different when *P* < 0.05 by ANOVA or Student's paired *t*-test.

### 2.3. Patient Selection, Clinical Assessment, and Electromyography Recordings

Eleven patients (6 females) with probable or definite ALS according to El Escorial criteria were recruited and analyzed at the ALS centre of Policlinico Umberto I, Sapienza University (Table 1). Inclusion criteria were as follows: male or female subjects older than 18 years; disease duration lower than 60 months; treatment with riluzole for at least 3 months before the study; no history of alcohol or substance abuse; no other neuromuscular disorders and diabetes mellitus; no marked lower limbs spasticity (>2 on the modified Ashworth scale); absence of psychiatric disorders or cognitive impairment at first evaluation; no concomitant therapy with psychoactive drugs.

Muscle strength of lower limbs was assessed with the Medical Research Council (MRC) score for muscle strength [26], an ordinal scale ranging from 0 (absence of movement) to 5 (normal contraction against full resistance) that quantifies muscle weakness in isolated muscles or muscle groups. Five muscle groups in lower limbs are tested: hip flexors, knee extensors, knee flexors, ankle plantar flexors, and ankle dorsiflexors.

An electromyographic instrument (System Plus Evolution 1.04.0104) employing a one-channel montage (sensitivity 50 *µ*V/division; low frequency filter 20 Hz; high frequency filter 5 KHz) was used to detect electrical activity. During the electrophysiological procedures, skin temperature was maintained between 31 and 34°C.

Monopolar needle electrodes were utilized to detect the insertional activity. Needle electromyographic signals were recorded from the right anterior tibialis muscle at a site approximating the demarcation between the proximal one-third and distal two-thirds of the muscle [27]. Three consecutive needle insertions (10 s each) with the same depth of insertion of needle (1.5 cm) per session were performed in all patients. A reference landmark by indelible pen allowed recording at the same muscle site in the second session. The number of Fibs discharges in each recording was counted manually.

Right tibialis anterior compound muscle action potential (CMAP) was evoked by peroneal nerve stimulation. The stimulating cathode was placed at the posterior-lateral area of the fibular head. The point for the recording surface electrodes was placed 8 cm from the cathode over the tibialis anterior. Reference electrodes were located over the tendons of the tibialis anterior. The latency of CMAP was measured from the stimulus artefact to the onset point, and the amplitude was determined from baseline to the highest negative peak [28].

Recordings were performed before and after a one-week suspension of riluzole treatment. Data were compared using the Wilcoxon signed rank test. Results were considered to be significantly different when *P* < 0.05.

The study was conducted in accordance with the guidelines of the* Declaration of Helsinki*.

## 3. Results

### 3.1. Effect of Riluzole on *I*_Na_ in ALS Myotubes

**Figure 1:**
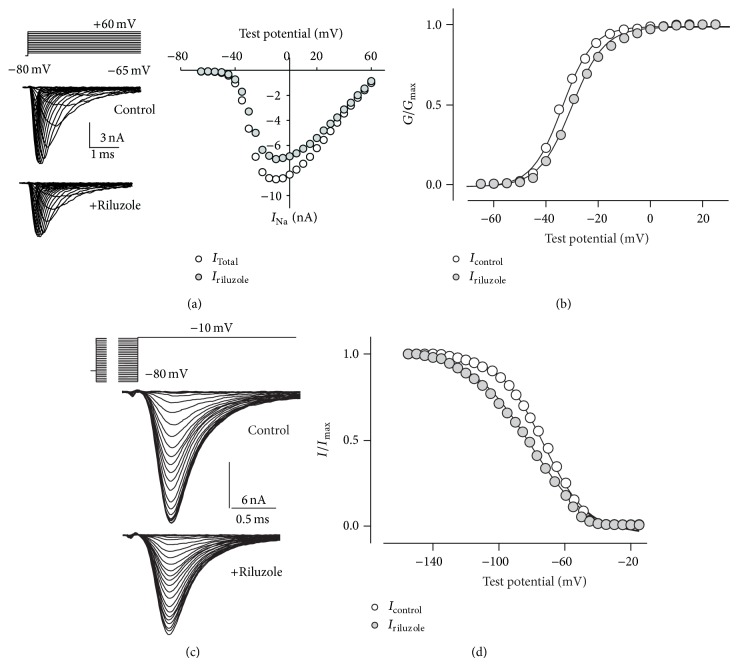
Effect of riluzole on voltage-gated sodium currents. (a) Family of currents evoked by test pulses from −65 mV to +60 mV in 5 mV increments (top traces), from a steady holding potential of −80 mV, under control conditions or in the presence of riluzole (1 *μ*M, 30 s pretreatment). Peak current-voltage plot was constructed for the same recordings. (b) Activation curves obtained in the absence or presence of riluzole, with the best fitting Boltzmann curves (black lines). Riluzole induced a depolarizing shift of the curve of about 3 mV and *k*_*A*_ was slightly but significantly increased. (c) Typical currents evoked by fast inactivation two-pulse protocol (top traces): a prepulse (20 ms) from −140 mV to −15 mV in 5 mV increments followed by a test pulse to −10 mV. Holding potential between recordings was −80 mV. (d) Steady-state fast inactivation curves had a hyperpolarizing shift, accompanied by a decrease of *k*_*I*_. Symbols as indicated.

Voltage-gated Na^+^ current (*I*_Na_) was recorded in all multinucleated myotubes (3–8 days differentiation) examined. Current density was 0.27 ± 0.03 nA/pF (*n* = 22) with the largest current obtained at test potentials of −20 to 0 mV (Figure 1(a)). Current was reduced to 28 ± 3% (*n* = 10) of control value by TTX (1 *μ*M, not shown), as expected in myotubes which express a mixture of Na_V_1.4 and Na_V_1.5 channels, with half-inhibitory TTX concentration of 10 nM and 3 *μ*M, respectively [23], thus confirming that recorded signals were* bona fide* Na^+^ currents.

Applying riluzole 1 *μ*M, a clinically relevant concentration [9], 30 s before stimulation, total *I*_Na_ amplitude was 79 ± 2% (*n* = 22) of untreated value (Figure 1(a)). In all cells tested, activation and steady-state fast inactivation curves were modified in the presence of riluzole. The activation curve had a depolarizing shift (Figure 1(b)), with a mean increase for *V*_*A*_ of 2.4 ± 0.3 mV (*n* = 22, *P* < 0.0004), and *k*_*A*_ was significantly increased by 0.6 ± 0.1 mV (*n* = 22, *P* = 0.0004). Steady-state fast inactivation curves had a hyperpolarizing shift (Figures 1(c) and 1(d)), with *V*_*I*_ decreasing by −8.7 ± 1.5 mV (*n* = 7, *P* = 0.001) and *k*_*I*_ by −3.5 ± 0.5 mV (*n* = 7; *P* = 0.0004).

### 3.2. Effect of Riluzole on Action Potentials

**Figure 2:**
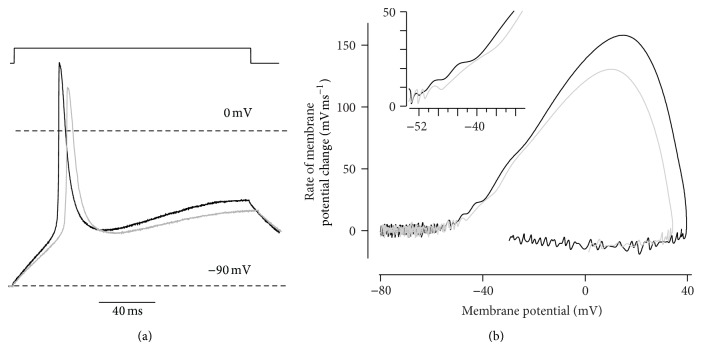
Effect of riluzole on action potentials. Action potentials (a) elicited by a depolarizing current (top, 0.2 nA) and phase plot (b) of the same traces. In the presence of riluzole (grey traces), both peak depolarization and rate of rise were reduced, in spite of unchanged holding potential. Magnification of the inflection point (inset) discloses a depolarizing shift of the threshold of spike onset. The inflection point represents the threshold potential, the positive peak corresponds to the maximal depolarization rate, and the largest value on the horizontal axis is the spike peak value.

These riluzole-induced shifts in activation and fast inactivation of *I*_Na_ might reverberate on action potential generation, reducing spike amplitude (because of enhanced fast inactivation) and rate of rise (due to reduced current amplitude). Small threshold modifications due to the depolarizing shift of channel activation were also expected. We therefore recorded action potentials evoked by depolarizing currents of increasing amplitude, using current clamp mode. In these myotubes, membrane resting potential ranged between −40 and −25 mV, so hyperpolarization (to −89.0 ± 1.4 mV, *n* = 5) was required to elicit action potentials. Depolarizing currents evoked overshooting spikes in all myotubes tested, but amplitude and rate of rise were reduced in the presence of riluzole (Figure 2(a)). In the five myotubes tested, spike peak and rate of rise were 29.7 ± 5.1 mV and 119 ± 18 mVms^−1^ under control conditions and 21.3 ± 5.3 mV and 89 ± 17 mVms^−1^, in the presence of riluzole (*P* ≤ 0.002 by paired Student's *t*-test). Action potential peak was delayed by 1.8 ± 0.7 ms (*n* = 5; *P* = 0.03). Furthermore, phase plots showed a small shift in threshold potential, compatible with the depolarizing shift of *V*_*A*_ measured in each myotube (Figure 2(b)).

Thus, riluzole reduces excitability of myogenic cells but does not prevent action potential generation in response to depolarizing stimuli, suggesting that it might be ineffective on muscle fibrillations. We therefore investigated this point directly.

### 3.3. Human Studies

**Figure 3:**
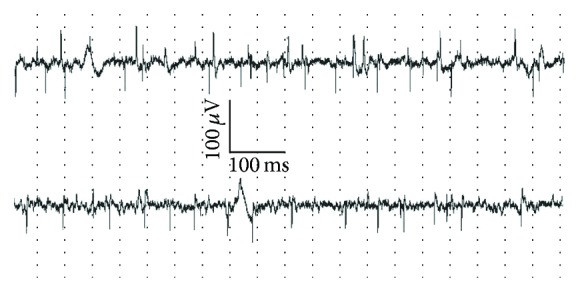
Fibrillation potentials in an ALS patient before and after riluzole suspension. Electromyography recordings performed in one ALS patient during riluzole therapy (top trace) and after a 1-week suspension of treatment (bottom trace). Notice that fibrillation potentials showed no significant change.

All patients were treated with riluzole (average treatment duration, 13.8 months) and suspended treatment for one week for this study. This time is adequate for riluzole washout, estimated to be complete within about 40 hours of withdrawal [29]. Multiple Fibs were detected in all patients' anterior tibialis muscle (Figure 3). Across the 3 insertions performed, their number had small fluctuations in all but Patients 5 and 7, which suggests that variability due to needle position is limited. In the second recording session, one week after riluzole withdrawal, clinical status and CMAP amplitude were unchanged for all patients (Table 1), indicating that no relevant alteration in muscle innervation took place. Fibs continued to be present in all patients, and variability between insertions did not change. When compared to the first recording session, the total number of Fibs changed by less than 25% in six patients; in the other 5, changes ranged between a 55% increase (Patient 2) and a 70% decrease (Patient 1). Overall, there was no statistically significant variation in the number of Fibs upon riluzole treatment suspension (*P* = 0.69) (Table 1). The effect of riluzole withdrawal showed no correlation with patients' age, sex, disease duration, or Fibs frequency at baseline (data not shown).

## 4. Discussion

In this paper we studied the effect of riluzole on voltage-gated sodium channels in human myotubes and on Fibs in ALS patients.* In vitro*, we show that riluzole at a therapeutically used concentration reduces *I*_Na_ and shifts the voltage dependence of gating. As a consequence, action potentials evoked by depolarizing currents have lower amplitude and reduced rate of rise. These results clearly indicate that riluzole acts on channels present on muscle fibres. The therapeutic implications of this nonneuronal effect, if any, are presently unknown. For instance, the documented partial block of muscle Na_V_ channels may result in dampened spontaneous activations of these channels and limit Fibs occurrence in patients' denervated muscle fibres.

Here we report that Fibs were consistently recorded during riluzole treatment in ALS patients, a clear indication that riluzole does not suppress muscle fibrillations. We were unable to detect univocal effects on Fibs upon riluzole withdrawal in the group of patients considered. Likely, other factors influence the frequency of Fibs, an inherently random process. Would a larger cohort of patients yield more conclusive data? The* in vitro* findings here reported indicate that riluzole has a small effect on action potential firing. Data obtained in patients do not disclose even a trend in the change of Fibs frequency upon riluzole withdrawal. Thus, extension of the* in vivo* study to a larger number of patients appears of little significance, also considering the discomfort of the procedure and the burden of repeated hospital access for heavily disabled patients.

None of the previous studies showing that Na_V_ channel blockers can suppress Fibs* in vivo* [5, 6] assessed the plasma concentration of the drugs, so it is not possible to correlate disappearance of Fibs with the extent of Na_V_ channels blockade. In organ cultures of rat muscle, TTX prevented Fibs when used at 1 *μ*M [4], which we show here to block a larger fraction of *I*_Na_ than riluzole at the concentration used. Thus, it is likely that riluzole-induced block of muscle *I*_Na_ is not sufficient to prevent Fibs. The aforementioned studies [4–6] report effects observed within minutes of drug application, whereas our data were collected in patients routinely taking riluzole or after a washout lasting one week, so that it cannot be ruled out that adaptations to long-term presence/absence of riluzole contribute to its inability to prevent Fibs in the examined patients.

A reduced rate of rise of evoked action potentials under riluzole, here reported for cultured myotubes, was also observed in guinea pig cardiac Purkinje fibres [30] but not in rat ventricular cardiomyocytes [31].

## 5. Conclusions

This study shows that riluzole at a typical clinical concentration partially blocks muscle Na_V_ channels and dampens—but does not abolish—the action potentials evoked by depolarizing stimuli in ALS myotubes. As suggested by* in vitro* inability to fully suppress spikes, riluzole does not prevent muscle Fibs in ALS patients, apparently having very limited impact on this symptom. Therefore, the pattern of Fibs disappearance during disease progression is unlikely to be directly influenced by riluzole-induced block of muscle Na_V_ channels.

## Figures and Tables

**Table 1:** Patients characteristics.

Patient (sex)	Age (years)	Disease duration (months)	Clinical onset	+Riluzole			−Riluzole		
				*N*_Fibs_	MRC right lower limb	CMAP peroneal nerve (mV)	*N*_Fibs_	MRC right lower limb	CMAP peroneal nerve (mV)
1 (M)	55	34	Spinal	81.3 (88, 80, 76)	19	2.6	24.7 (28, 30, 16)	20	2.5
2 (F)	53	24	Spinal	94.7 (100, 90, 94)	21	2.8	146.7 (145, 140, 155)	20	2.9
3 (F)	53	23	Spinal	133.3 (148, 140, 112)	21	3.1	73.3 (70, 70, 80)	21	3.0
4 (M)	71	36	Spinal	126.6 (115, 127, 138)	12	0.10	153.3 (158, 140, 162)	12	0.14
5 (M)	56	55	Spinal	60 (85, 56, 39)	18	1.8	48.3 (43, 35, 67)	18	1.7
6 (F)	61	30	Spinal	106.7 (105, 102, 113)	25	3.8	124.7 (118, 128, 128)	25	3.9
7 (F)	51	19	Spinal	63.3 (55, 47, 88)	26	4.0	48.3 (64, 49, 32)	26	4.2
8 (M)	58	16	Bulbar	27 (30, 25, 26)	31	9.7	32.3 (34, 38, 25)	31	9.8
9 (F)	75	27	Bulbar	32.7 (28, 37, 33)	27	9.8	36 (38, 32, 38)	27	9.6
10 (F)	63	10	Spinal	82 (78, 88, 80)	30	8.9	40 (45, 40, 35)	30	8.7
11 (M)	50	13	Spinal	106.7 (101, 100, 119)	29	7.7	33.3 (36, 35, 29)	29	7.6

Demographic and clinical characteristics of the 11 patients with ALS evaluated during riluzole treatment (+Riluzole) and after 1-week withdrawal (−Riluzole). *N*_Fibs_: average number (number in each insertion) of fibrillation potentials (Fibs); MRC lower limbs: Medical Research Council score for lower limbs; CMAP: amplitude of peroneal compound muscle action potential. Number of Fibs is not significantly affected by riluzole treatment (*P* = 0.69 by Wilcoxon test).
